# Accuracy and reproducibility of a large language model in assessing spirometry test acceptability and interpretation: a performance study

**DOI:** 10.3389/fmed.2026.1974948

**Published:** 2026-10-14

**Authors:** Hajed M. Al-Otaibi, Husam I. Alahmadi, Malik A. Althobiani, Kannan Sridharan, Abdullah A. Alqarni, Ghazi A. Alotaibi

**Affiliations:** 1 Department of Respiratory Therapy, Faculty of Medical Rehabilitation Sciences, King Abdulaziz University, Jeddah, Saudi Arabia; 2 Respiratory Therapy Unit, King Abdulaziz University Hospital, King Abdulaziz University, Jeddah, Saudi Arabia; 3 Department of Pharmacology & Therapeutics, College of Medicine and Health Sciences, Arabian Gulf University, Manama, Bahrain; 4 Arabian Gulf University, Manama, Bahrain; 5 Imam Abdulrahman Bin Faisal University, Dammam, Saudi Arabia

**Keywords:** acceptability, AI systems, chatGPT, large language model, spirometry

## Abstract

**Background:**

Spirometry is one of the most used pulmonary function tests for assessing respiratory function and diagnosing ventilatory disorders in clinical practice. Evaluation of test validity and interpretation is typically performed by trained healthcare professionals. The present study aims to evaluate the capability of a publicly available large language model (LLM) to perform quality assurance and interpretations of spirometry tests.

**Methods:**

A total of 150 spirometry tests were retrospectively collected from the pulmonary function laboratories. The spirometry tests were evaluated for acceptability and interpreted by clinicians according to ATS/ERS recommendations as part of standard clinical practice. Subsequently, a publicly available LLM (ChatGPT 5.5) was prompted to evaluate the acceptability and interpret the same spirometry tests according to ATS/ERS criteria. The model interpreted each spirometry test on three separate occasions for assessing reproducibility. Accuracy, sensitivity, specificity, positive predictive value (PPV), and negative predictive value (NPV), with 95% confidence intervals (CIs), were calculated by comparing LLM assessments with clinician assessments of spirometry test acceptability and interpretation.

**Results:**

For spirometry acceptability evaluation, ChatGPT demonstrated an overall accuracy of 87% (95% CI: 81%-92%), sensitivity of 91% (95% CI: 85%-95%), specificity of 61% (95% CI 39%-80%), PPV of 94% (95% CI: 89%-97%), and NPV of 48(95% CI: 29%-67%) with Cohen's k of 0.46. The spiromteric interpretation agreement with the clinicians reference standard was moderate, with exact agreement of 0.61 (95% CI: 0.54–0.69) while Cohen's Kappa (Unweighted) was 0.47 (95% CI: 0.37–0.56) and Macro F1 score was 0.39 (95% CI: 0.33–0.49). Reproducibility across three independent trials was moderate (Fleiss’ *κ* 0.58). Class-specific interpretation performance varied by category; sensitivity was highest for normal pattern and restrictive defects (0.72), whereas sensitivity for obstructive defects was 0.27. F1-scores were highest for normal and restrictive defects (0.81 and 0.62, respectively).

**Conclusion:**

The current findings suggest that ChatGPT 5.5 has a limited capability, at its current stage of development, to serve as a reliable tool for independent spirometry interpretation in clinical practice. Nevertheless, it may serve as an adjunct to clinical decision-making and educational tools. Prospective controlled studies are needed to confirm these findings.

## Introduction

Spirometry is one of the most widely used pulmonary function tests in clinical settings. It is mainly used to diagnose and monitor pulmonary disorders, evaluate respiratory symptoms, and support preoperative and pre-employment assessments (1). It provides physiological measurements of changes in lung volume over time and airflow in relation to lung volume. Spirometry test is mainly effort-dependent; subject has to perform maximal effort to produce valid results. It has been shown that spirometry tests that do not meet the standards of test quality may mislead clinicians and result in misclassification (2).

American Thoracic Society (ATS) recognized the importance of the spirometry tests validity more than 40 years ago (3). Therefore, ATS has established several recommendations to standardize the procedures and ensure validity of the tests. These recommendations were updated several times (2, 4–6). The aim of spirometry standardization is to ensure accurate, reliable, and repeatable results regardless of differences in location, operator or device type (6). Enhancing spirometry test quality supports consistent reporting and interpretation, which ultimately improves patient care. Therefore, clinicians should evaluate the spirometry tests and judge their acceptability before proceeding to interpretation. However, early reports found that considerable proportion of spirometry tests fail to meet standardization criteria (7). Similarly, van de Hei et al. (2020) reported that clinicians accepted a large proportion of spirometry tests, although only a small proportion actually met the ATS/European Respiratory Society (ERS) acceptability criteria and the agreement on diagnosis between assessors was only fair to moderate (*κ* 0.39–0.44) (8). Despite being conducted by experienced personnel, Cengiz et al. (2025) found that only 62.5% of 1,000 spirometry tests fulfilled the ATS/ERS 2019 acceptability criteria, underscoring the persistent challenges in achieving optimal spirometry quality in clinical practice (9).

Artificial intelligence (AI) programs are rapidly emerging across various medical specialties. Accumulating evidence supports their substantial potential to enhance clinical practice and decision-making. Rajpurkar et al. (2018) demonstrated that a well-trained deep learning model achieved performance comparable to certified radiologists across multiple thoracic pathologies on chest radiographs (10). Similarly, AI models have been reported to achieve high accuracy comparable to radiologists in interpreting chest radiographs for the detection of common thoracic pathologies (11). Iqbal et al. (2024) concluded that artificial intelligence and deep learning programs show promising clinical utility in the interpretation of chest radiographs and in detecting a wide spectrum of pulmonary pathologies (12). Similarly, Hannun et al. (2019) found that a deep learning model was capable of achieving high sensitivity and specificity in detecting ECG arrhythmias, with performance comparable to certified cardiologists (13). Dermatologists evaluated a deep convolutional neural network for the classification of skin cancer images and reported performance comparable to certified dermatologists (14).

Das et al. (2020) developed convolutional neural network to evaluate the acceptability and usability of spirometry tests. The model shows comparable agreement with certified reviewers and were able to detect common spirometric technical errors (15). Slimily, it has shown that a learning algorithm was able to achieve satisfactory level to identify acceptable spirometry tests and captures the common technical errors (16). Likewise, it was shown that the deep learning model was able to reliably evaluate spirometry acceptability using both curve morphology and spirometric indices (17). In a systematic review, Lopez-Cany et al. (2025) concluded that AI-based models have the potential to help clinicians in evaluating the validity and reliability of spirometry tests. It also has the potential to address human related variability. However, the authors stated that the studies are scarce and called for further evaluation of AI tools (18).

The aim of the present study is to evaluate the capability of a publicly available large language model (LLM) to perform satisfactory quality assurance of spirometry tests according to ATS/ERS standards. It also aims to evaluate its capability to interpret spirometry results in comparison with pulmonary physicians.

## Methods

### Study design and setting

A total of 150 spirometry tests were retrospectively collected from two pulmonary function testing laboratories at two different hospitals in Jeddah, Saudi Arabia. These tests were performed during the period from 2022 to 2026. Each participating hospital used its respective reference equations for the calculation of predicted spirometric values. The Global Lung Function Initiative reference equations were used at one hospital, while the European Community for Coal and Steel reference equations were used at the other. The reference values were calculated using the appropriate population-specific reference equations as per standard of care. Spirometry tests were included if their acceptability and repeatability had been assessed by the pulmonary team and classified as either acceptable or unacceptable. A final clinical interpretation was available only for tests deemed acceptable. Tests were excluded if they are unreadable, patient demographics information is unavailable, and there is no interpretation. There was no intention to select spirometry tests based on patient demographics, clinical diagnosis, or the morphology of the spirometry curves. Tests were included irrespective of these characteristics, provided they met the predefined inclusion criteria. Spirometry tests were performed by experienced pulmonary function laboratory staff with more than 10 years of pulmonary function testing experience. The sample was considered sufficient to provide preliminary estimates of accuracy, agreement, sensitivity, specificity, and reproducibility.

The study was approved by the King Abdulaziz University Hospital Research Ethics Committee (reference no. 135-26) as a non-interventional study of existing clinical records. Before upload to the LLM platform, all spirometry records were de-identified through removal of patient names and medical record numbers.

### Spirometry data and reference standard

Spirometry reports included participant demographic information (age, sex, height, weight, ethnicity, and smoking status), together with baseline spirometric measurements and, when available, post-bronchodilator measurements. The reports also included flow-volume loops, volume-time curves, and numerical spirometric indices.

The technical quality of each spirometry test was independently assessed by a senior respiratory therapist, who is a certified pulmonary function technologist with extensive experience in pulmonary function testing, based on the (ATS/ERS) spirometry criteria (6). The assessed outcomes were overall acceptability, satisfactory start of test, minimum exhalation time, and end-of-forced-expiration criteria. According to the study protocol, a test was considered acceptable when all three individual quality criteria were fulfilled. Thus, failure to meet any one of these criteria rendered the entire test unacceptable. Subsequently, unacceptable spirometry tests were categorized as uninterpretable and included alongside the other predefined spirometric patterns in the interpretation analysis. Accordingly, these spirometry tests remained classified as “uninterpretable” in the reference standard and were retained in the study. This category was included to assess the capability of the LLM to correctly identify spirometry tests that did not meet the predefined acceptability criteria and were therefore unsuitable for further interpretation. The recorded respiratory therapist assessment served as the reference standard for analyses of overall acceptability and the individual quality criterion.

The reference interpretation for each test was assigned by one of the pulmonary team according to standard spirometry interpretation principles and ATS/ERS guidance (6) as part of routine patients’ care. These assessments served as the reference standard for analyses of interpretation and post-bronchodilator responsiveness evaluation.

### LLM index test and prompting procedure

The LLM was prompted to extract all patients demographics which include gender, race, age, height, weight, body mass index, and smoking status for all patients. Baseline spirometry variables were extracted from each test, including the measured and predicted values for forced vital capacity (FVC), forced expiratory volume in one second (FEV₁), FEV₁/FVC ratio, forced expiratory flow between 25% and 75% of FVC (FEF₂₅-₇₅_%_), and peak expiratory flow rate (PEFR). The percentage of predicted value for each parameter was calculated as (measured value/predicted value) × 100. Similarly, when available, post-bronchodilator spirometry data were extracted, including the measured values, predicted values, and percentage of predicted values for FVC, FEV₁, FEV₁/FVC ratio, FEF₂₅-₇₅_%_, and PEFR. The percentage of predicted value for each parameter was calculated as (measured value/predicted value) × 100. Extracted data were reviewed for completeness and internal consistency, and missing values were recorded without imputation.

The index test was ChatGPT, based on GPT-5.5 (19). A standardized instructions set and Ruppel's Manual of Pulmonary Function Testing, 12th edition (1), were provided to the LLM. De-identified spirometry reports were then uploaded individually. The LLM was instructed to evaluate each spirometry test using the available flow-volume curve, volume-time curve, numerical spirometric data, and the provided instructions.

For technical quality assessment, the LLM was prompted to classify overall acceptability and the following individual criteria as Yes or No: satisfactory start of test, minimum exhalation time, and end-of-forced-expiration criteria. If any one of the three acceptability criteria was not met, the LLM was instructed to classify the spirometry test as unacceptable. Such tests were considered invalid for interpretation and were therefore assigned as “uninterpretable” in the next state (interpretation step).

For spirometry interpretation, the LLM was instructed to assign one of the following six categories to each individual spirometry test: spirometry within normal limits, obstructive ventilatory defect, restrictive ventilatory defect, combined ventilatory defect, nonspecific ventilatory defect, or uninterpretable. Spirometric patterns were classified according to established ATS/ERS interpretive criteria, as described in Ruppel's Manual of Pulmonary Function Testing and the ERS/ATS technical standard for interpretive strategies for routine lung function tests (1). Spirometry within normal limits was defined as FVC and FEV₁/FVC at or above their respective lower limits of normal (LLN). Obstruction was defined as an FEV₁/FVC below the LLN. A possible restrictive pattern was defined as a reduced FVC with a preserved FEV₁/FVC. A mixed pattern was defined as reductions in both FEV₁/FVC and FVC, while a nonspecific pattern was defined as a reduced FEV₁ and/or FVC with a preserved FEV₁/FVC, without confirmed restriction. For tests with available post-bronchodilator measurements, the LLM was instructed to classify bronchodilator responsiveness as significant or non-significant, using an increase in FEV_1_ and/or FVC of more than 10%.

The LLM was provided with the above instructions and the relevant chapters from Ruppel's Manual of Pulmonary Function Testing, which were uploaded as reference documents. After uploading these materials, the following prompt was entered: Strictly according to the attached instructions and Ruppels manual of pulmonary function testing perform interpretation as one of six categories to the following spirometry tests. Following submission of the prompt, the spirometry test reports were uploaded individually for analysis.

Each spirometry test was evaluated on three different occasions using the same standardized prompt, input data, and the same page. The initial assessment was defined as Trial 1, and the two repeated assessments were defined as Trials 2 and 3.

### Harmonization of interpretation categories

Before analysis, clinician and LLM interpretation labels were harmonized into six mutually exclusive categories: normal, obstructive, restrictive, mixed, nonspecific, and uninterpretable. “Combined ventilatory defect” and “mixed ventilatory defect” were harmonized as mixed. “Fixed airway obstruction” was harmonized as obstructive. Labels denoting normal spirometry or spirometry within normal limits were harmonized as normal. Spelling or formatting variants of nonspecific ventilatory defect were harmonized as nonspecific. Tests labelled as uninterpretable by either source were categorized as uninterpretable according to the original reported label.

The primary analysis of spirometry interpretation used the LLM result from Trial 1. Trials 2 and 3 were used exclusively for reproducibility analyses.

### Bronchodilator reversibility

Bronchodilator responsiveness was assessed among the spirometry tests with available pre- and post-bronchodilator measurements and valid paired classification. Participants whose responsiveness was recorded as “Not assessed” by either the LLM or the clinicians were excluded from this analysis. Bronchodilator response was calculated as [(post−pre) ÷ predicted value] × 100. The 2022 ERS/ATS standard defines a positive response as >10% of predicted FEV₁ or FVC (20). The clinician responsiveness assessment served as the reference standard.

### Statistical analysis

Participant characteristics and spirometry measurements were summarized using mean ± standard deviation and range for continuous variables, and frequency with percentage for categorical variables.

For overall spirometry acceptability and each individual quality criterion, the LLM was compared with the respiratory therapist reference standard using 2 × 2 contingency tables. The positive class was acceptability or fulfillment of the relevant quality criterion. Accuracy, sensitivity, specificity, positive predictive value (PPV), and negative predictive value (NPV) were calculated with 95% confidence intervals using the Wilson score method. Unweighted Cohen's *κ* was calculated to quantify agreement beyond chance.

For multicategory spirometry interpretation, agreement between the Trial 1 LLM results and the reference standard was assessed using exact agreement, unweighted Cohen's *κ*, and macro F1-score. Exact agreement and macro F1-score were calculated across the six harmonized interpretation categories. Ninety-five percent confidence intervals for aggregate interpretation measures were estimated using 1,000 bootstrap resamples.

Class-specific performance was assessed using sensitivity, PPV, and F1-score for each clinician reference-standard category. Wilson score 95% confidence intervals were calculated for sensitivity and PPV. Estimates for categories with fewer than five reference-standard observations were considered exploratory and interpreted cautiously.

Reproducibility across the three LLM trials was assessed using pairwise percent agreement and unweighted Cohen's *κ* for Trial 1 versus Trial 2, Trial 2 versus Trial 3, and Trial 1 versus Trial 3. Overall agreement across all three trials was quantified using Fleiss’ *κ*.

For bronchodilator responsiveness, the LLM was compared with the clinician's reference standard using accuracy, sensitivity, specificity, PPV, NPV, and unweighted Cohen's *κ*. Wilson score 95% confidence intervals were calculated for interpretation performance measures.

Analyses were conducted using Python in Excel Microsoft® Excel® for Microsoft 365 MSO (Version 2608 Build 16.0.20321).

## Results

### Study cohort

A total of 150 participants were included in the final analytic cohort. Participant demographics and baseline spirometry are summarized in Table 1. The race category recorded in the pulmonary function report for selection of reference equations was Caucasian in 98.7% of participants, and 58.0% were female, with a mean age of 47.5 ± 18.2 years and mean BMI of 29.5 ± 6.5 kg/m^2^. Post-bronchodilator measurements were available for 134 participants (89.3%), of whom 117 had valid paired clinician and LLM bronchodilator responsiveness classifications and were included in the responsiveness analysis. Figure 1 illustrates the study flow diagram.

**Table 1:** Participant characteristics and spirometry measurements.

Characteristic	Overall (*N* = 150)	Range (min–max)
**Participant characteristics**		
Age, years	47.5 ± 18.2	12.0–84.0
Male sex, *n* (%)	63 (42.0)	—
Female sex, *n* (%)	87 (58.0)	—
Race: Caucasian, *n* (%)	148 (98.7)	—
Race: Black, *n* (%)	1 (0.7)	—
Race: Asian, *n* (%)	1 (0.7)	—
Height, cm	162.1 ± 8.8	140.0–187.0
Weight, kg	77.6 ± 19.0	23.8–142.3
Body mass index, kg/m^2^	29.5 ± 6.5	12.1–49.8
Smoker, *n* (%)	3 (2.0)	—
**Baseline spirometry**		
FVC, L	2.8 ± 0.9	1.0–5.6
FVC predicted, L	3.6 ± 0.8	1.8–5.6
FVC, % predicted	79.7 ± 17.6	32.0–121.0
FEV_1_, L	2.3 ± 0.8	0.6–4.5
FEV_1_ predicted, L	2.8 ± 0.7	1.1–4.6
FEV_1_, % predicted	80.9 ± 19.6	30.0–123.0
FEV_1_/FVC ratio	0.80 ± 0.10	0.37–0.99
FEV_1_/FVC predicted ratio	0.77 ± 0.04	0.68–0.90
FEV_1_/FVC, % predicted	103.2 ± 12.7	52.0–127.0
FEF_25%–75%_, L/s	2.4 ± 1.2	0.2–5.4
FEF_25%–75%_ predicted, L/s	3.2 ± 0.8	1.3–5.1
FEF_25%–75%_, % predicted	75.8 ± 32.6	10.0–168.0
PEF, L/s	6.3 ± 2.1	2.1–12.9
PEF predicted, L/s	6.9 ± 1.5	4.4–10.2
PEF, % predicted	91.2 ± 22.0	37.0–157.0
**Post-bronchodilator spirometry**		
FVC post-bronchodilator, L (*n* = 134)	2.9 ± 0.9	1.0–5.5
FEV_1_ post-bronchodilator, L (*n* = 134)	2.4 ± 0.8	0.7–4.3
FEF_25%–75%_ post-bronchodilator, L/s (*n* = 134)	2.7 ± 1.2	0.3–5.8
PEF post-bronchodilator, L/s (*n* = 134)	6.6 ± 2.0	2.0–11.9

Data are presented as mean ± standard deviation and range (minimum–maximum) for continuous variables and *n* (%) for categorical variables. Post-bronchodilator measurements were available for the indicated number of participants. BMI, body mass index; FEV_1_, forced expiratory volume in 1 s; FVC, forced vital capacity; FEF_25%–75%_, forced expiratory flow between 25% and 75% of forced vital capacity; PEF, peak expiratory flow.

**Figure 1:**
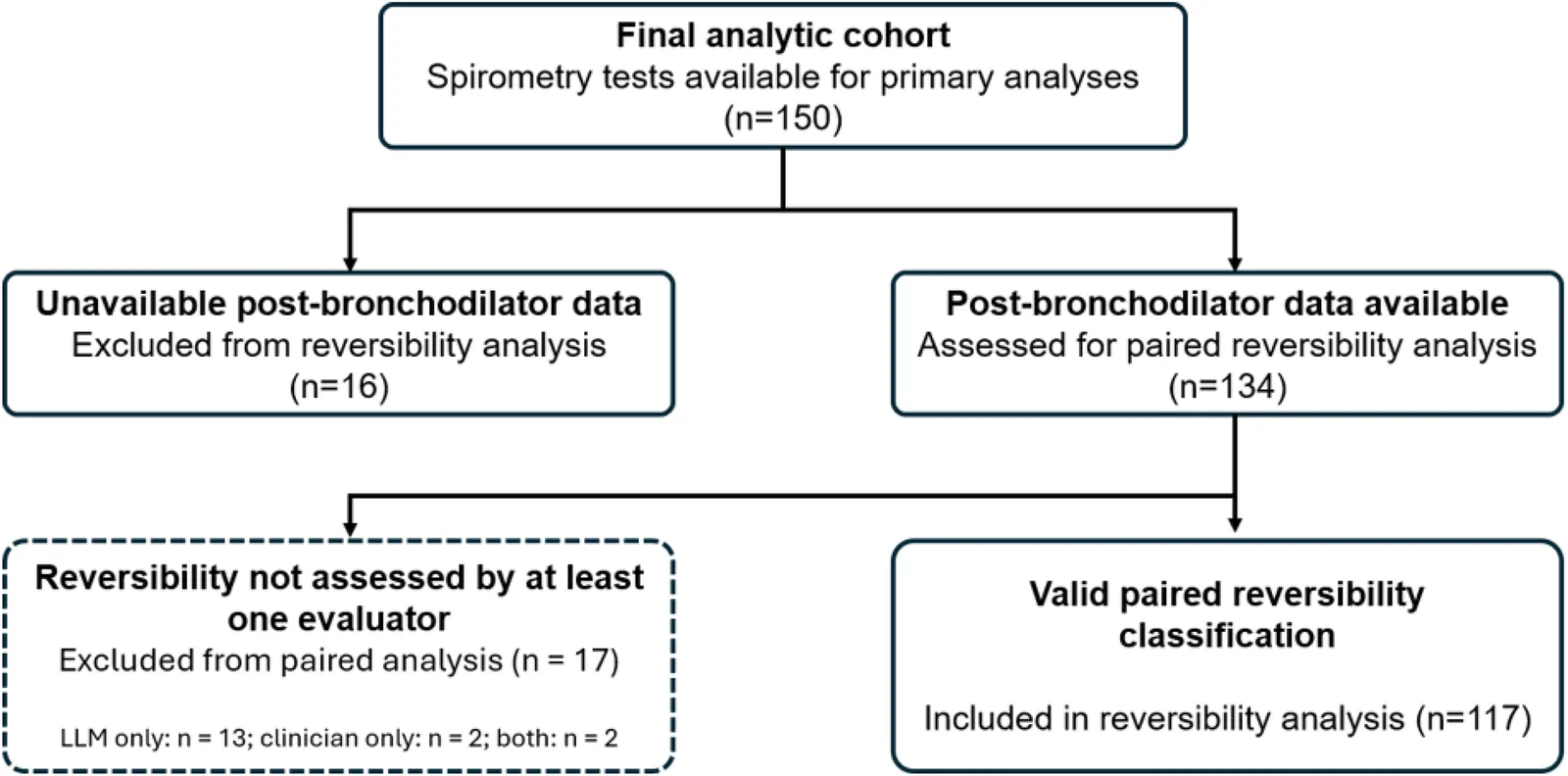
Study flow diagram illustrating the inclusion of 150 spirometry tests in the primary analyses of acceptability, interpretation, and reproducibility. Post-bronchodilator measurements were available for 134 participants, with 16 excluded from the bronchodilator responsiveness analysis due to missing measurements. Among the remaining 134 participants, 17 were excluded because poor test quality prevented responsiveness assessment by the LLM only (*n* = 13), the clinician only (*n* = 2), or both (*n* = 2). Consequently, 117 participants had valid paired bronchodilator responsiveness classifications and were included in the final responsiveness analysis.

### Technical quality and acceptability

The LLM demonstrated high accuracy for assessment of individual spirometry quality criteria with overall accuracy for spirometry acceptability of 0.87 (95% CI, 0.81–0.92). Sensitivity was high for overall acceptability (0.91; 95% CI, 0.85–0.95) and was near perfect across all criteria (≥0.99), whereas specificity was lower, particularly for the start-of-test criterion (0.25; 95% CI, 0.07–0.59). Agreement beyond chance was moderate for overall acceptability (Cohen's *κ* = 0.46) and start of test (Cohen's *κ* = 0.34), and substantial for exhalation time and end of forced expiration (Cohen's *κ* = 0.61 for both). Table 2 presents the performance of LLM for spirometry overall acceptability and individual quality criteria.

**Table 2:** Performance of the LLM for spirometry acceptability and individual quality criteria, using clinician assessment as the reference standard.

Outcome	N	Accuracy (95% CI)	Sensitivity (95% CI)	Specificity (95% CI)	PPV (95% CI)	NPV (95% CI)	Cohen's *κ*
Overall acceptability	150	0.87 (0.81, 0.92)	0.91 (0.85, 0.95)	0.61 (0.39, 0.80)	0.94 (0.89, 0.97)	0.48 (0.29, 0.67)	0.46
Start of test	150	0.95 (0.91, 0.98)	0.99 (0.96, 1.00)	0.25 (0.07, 0.59)	0.96 (0.91, 0.98)	0.67 (0.21, 0.94)	0.34
Exhalation time	150	0.94 (0.89, 0.97)	1.00 (0.97, 1.00)	0.47 (0.26, 0.69)	0.94 (0.88, 0.97)	1.00 (0.68, 1.00)	0.61
End of forced expiration	150	0.93 (0.88, 0.96)	1.00 (0.97, 1.00)	0.47 (0.27, 0.68)	0.93 (0.87, 0.96)	1.00 (0.70, 1.00)	0.61

The clinician assessment served as the reference standard. The positive class was acceptability/criterion fulfillment (Yes). Proportion confidence intervals were calculated using the Wilson score method. Cohen's κ values are unweighted. PPV, positive predictive value; NPV, negative predictive value; CI, confidence interval.

### Patterns of agreement in spirometry quality assessment

The confusion matrices for overall acceptability and the individual quality criteria are shown in Figure 2. Disagreements predominantly reflected LLM classification of clinician-rated unsatisfactory tests as satisfactory. False-negative classifications occurred for overall acceptability (*n* = 12), and start of test (*n* = 1), whereas no false-negative classifications occurred for exhalation time, or end of forced expiration. False-positive classifications were most frequent for end of forced expiration (*n* = 10) and exhalation time (*n* = 9), overall acceptability (*n* = 7) and start of test (*n* = 6).

**Figure 2:**
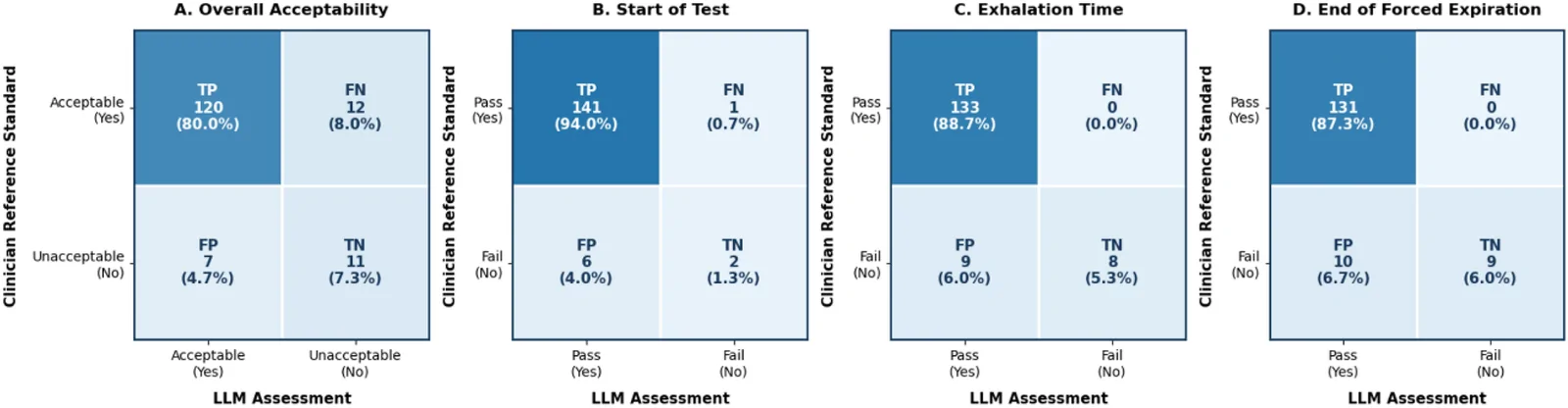
Confusion matrices for LLM assessment of overall spirometry acceptability and individual quality criteria compared with the clinician reference standard (*N* = 150). **(A)** Overall acceptability; **(B)** start of test; **(C)** exhalation time; (**D**) end of forced expiration. In each panel, rows represent the clinician reference standard, and columns represent the LLM assessment. Values indicate the number and percentage of all tests in each cell. Colour intensity reflects the proportion of tests in each cell. TP, true positive; FP, false positive; TN, true negative; FN, false negative; LLM, large language model.

### Interpretation performance and spirometric pattern classification

Aggregate multicategory agreement between the LLM and clinician reference standard was moderate, with an overall exact agreement of 0.61 (95% CI, 0.54–0.69) and an unweighted Cohen's *κ* of 0.47 (95% CI, 0.37–0.56) (Table 3). The macro F1-score across the six harmonized interpretation categories was 0.39 (95% CI, 0.33–0.49).

**Table 3:** Aggregate performance and multicategory agreement of the LLM compared with the clinician reference standard for spirometry interpretation.

Metric	Value (95% CI)
Overall Exact Agreement	0.61 (0.54–0.69)
Cohen's Kappa (Unweighted)	0.47 (0.37–0.56)
Macro F1-Score	0.39 (0.33–0.49)

Values are presented as the point estimate with 95% confidence intervals (CI) in parentheses. All metrics were calculated using the harmonized interpretation categories (Normal, Obstructive, Restrictive, Mixed, Nonspecific, and Uninterpretable) based on the first interpretation trial (Trail 1). Confidence intervals were derived using 1,000 bootstrap iterations. Macro F1-score represents the arithmetic mean of F1-scores across all classes, providing equal weight to each category regardless of frequency.

Class-specific performance varied across interpretation categories (Table 4; Figure 3). Sensitivity was highest for normal and restrictive defects, at 0.72 for both categories. The model identified obstructive defects less reliably, with a sensitivity of 0.27 (95% CI, 0.14–0.46). PPV was highest for normal spirometry (0.91; 95% CI, 0.81–0.96). Correspondingly, the F1-score was highest for normal spirometry (0.81), followed by restrictive spirometry (0.62), whereas performance was lower for obstructive (0.39) and uninterpretable classifications (0.50). Estimates for mixed and nonspecific categories were exploratory because of sparse clinician reference-standard observations.

**Table 4:** Class-specific interpretation performance of the LLM for spirometry interpretation compared with the clinician reference standard (*N* = 150).

Interpretation Category	N (Reference)	TP/N	Sensitivity (95% CI)	PPV (95% CI)	F1-Score
Normal	72	52/72	0.72 (0.61–0.81)	0.91 (0.81–0.96)	0.81
Obstructive	26	7/26	0.27 (0.14–0.46)	0.70 (0.40–0.89)	0.39
Restrictive	32	23/32	0.72 (0.55–0.84)	0.55 (0.40–0.69)	0.62
Mixed^a^	1	0/1	0.00 (0.00–0.79)	0.00 (0.00–0.18)	0.00
Nonspecific^a^	2	0/2	0.00 (0.00–0.66)	0.00 (0.00–0.00)	0.00
Uninterpretable	17	10/17	0.59 (0.36–0.78)	0.43 (0.26–0.63)	0.50

Clinician assessment served as the reference standard. Interpretation categories were harmonized prior to analysis. 95% confidence intervals were calculated using the Wilson score method.

a Indicates categories with sparse observations (*N* < 5) where performance estimates should be interpreted with caution. F1-score is the harmonic mean of sensitivity and positive predictive value (PPV). TP/N = true positives/number of clinician-diagnosed cases.

**Figure 3:**
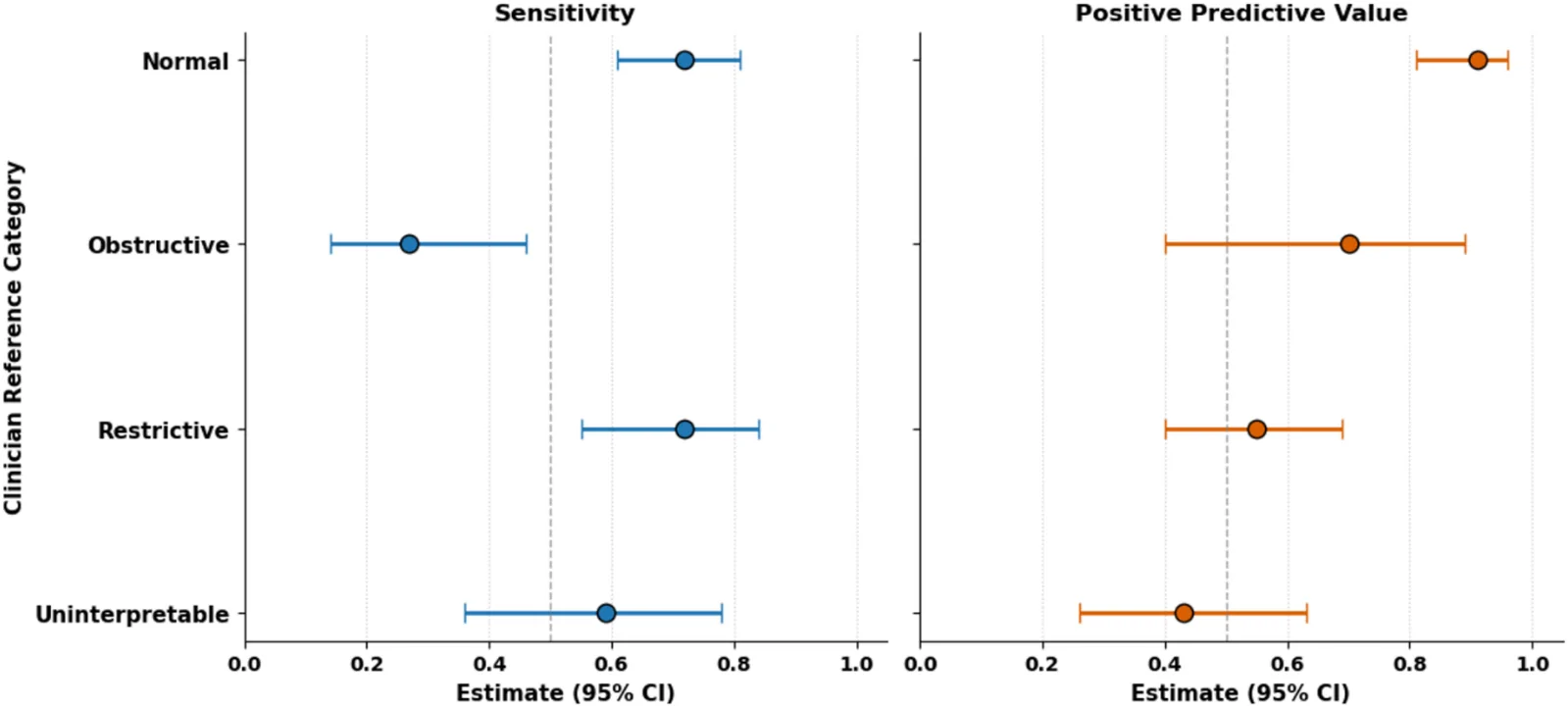
Class-specific interpretation performance of the LLM for spirometry interpretation. Points indicate sensitivity (left panel, blue) and positive predictive value (right panel, orange), and horizontal bars indicate 95% confidence intervals. The dashed vertical line at 0.5 is included as a visual reference. Mixed and nonspecific ventilatory defect categories were excluded because of sparse clinician reference-standard observations (*n* < 5). PPV, positive predictive value; CI, confidence interval.

Patterns of spirometric classification changes between clinician and LLM interpretations are illustrated in Figure 4. The figure displays transitions from clinician-assigned interpretation categories to LLM classifications in Trial 1. Exact agreement was most frequent for normal, restrictive, and uninterpretable classifications. No true-positive classifications were observed for mixed or nonspecific ventilatory defects; however, these categories had sparse reference-standard observations.

**Figure 4:**
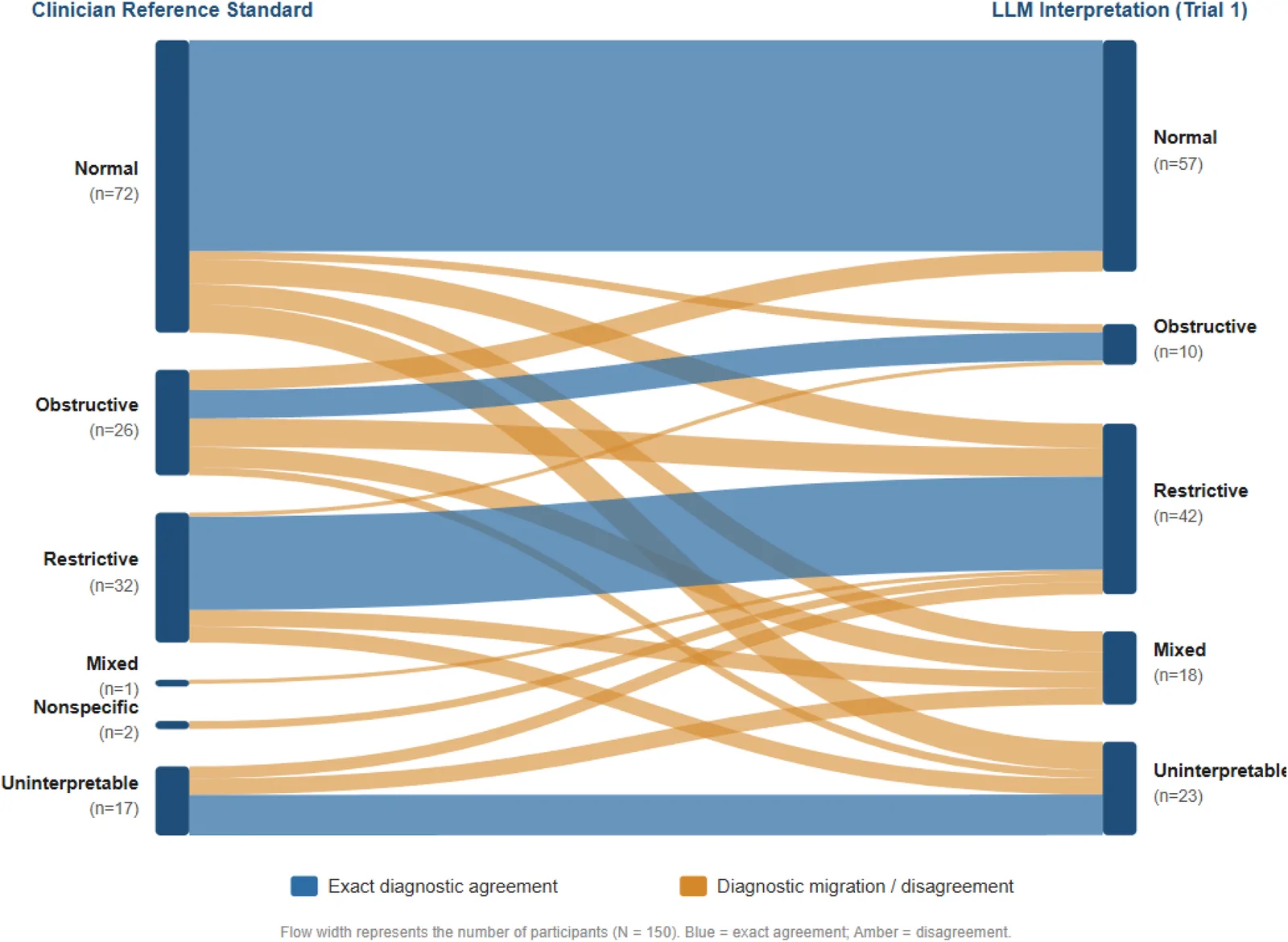
Migration of spirometry interpretations between the clinician reference standard and the LLM in the first trial (*N* = 150). Connecting flows represent transitions from clinician-assigned interpretation categories on the left to LLM-assigned categories on the right. Flow widths are proportional to the number of spirometry tests. Blue flows indicate exact agreement, whereas amber flows indicate disagreement. Interpretation categories were harmonized before comparison.

### Reproducibility and responsiveness assessment

The LLM showed a moderate overall reproducibility across three independent trials, with an overall Fleiss’ *κ* of 0.58. Pairwise agreement ranged from 61.3% to 79.3%, with Cohen's *κ* values ranging from 0.49 to 0.71. Table 5 presents reproducibility of LLM spirometry interpretation across the three independent trails.

**Table 5:** Reproducibility of LLM spirometry interpretations across three independent trials (*N* = 150).

Comparison	N	Percent Agreement	Cohen's/Fleiss’ κ
Trial 1 vs. Trial 2	150	66.0%	0.55
Trial 2 vs. Trial 3	150	79.3%	0.71
Trial 1 vs. Trial 3	150	61.3%	0.49
Overall reproducibility (Fleiss’ κ)	150	—	0.58

Trial 1 was the initial LLM interpretation; Trials 2 and 3 were repeated LLM interpretations of the same spirometry data. Pairwise agreement was assessed using percent agreement and Cohen's κ. Overall agreement across all three trials was assessed using Fleiss’ κ. Analyses used harmonized interpretation categories.

For bronchodilator responsiveness assessment, the LLM demonstrated an accuracy of 0.97 (95% CI, 0.93–0.99) and a specificity of 1.00 (95% CI, 0.96–1.00). Sensitivity for identifying significant post-bronchodilator responsiveness was 0.77 (95% CI, 0.50–0.92). PPV was 1.00, and NPV was 0.97. Agreement beyond chance was strong (Cohen's *κ* = 0.86). Table 6 presents performance of the LLM for assessment of post-bronchodilator responsiveness compared with the clinician reference standard.

**Table 6:** Performance of the LLM for assessment of post-bronchodilator responsiveness compared with the clinician reference standard (*n* = 117).

Diagnostic Metric	Value (95% CI) [*n* = 117]
Accuracy	0.97 (0.93–0.99)
Sensitivity	0.77 (0.50–0.92)
Specificity	1.00 (0.96–1.00)
Positive Predictive Value	1.00 (0.72–1.00)
Negative Predictive Value	0.97 (0.92–0.99)
Cohen's Kappa	0.86

Clinician assessment served as the reference standard. The paired responsiveness analysis included 117 participants with available post-bronchodilator measurements and valid clinician and LLM post-bronchodilator responsiveness classifications. Sixteen participants had no post-bronchodilator measurements, and 17 participants with post-bronchodilator data were excluded because post-bronchodilator responsiveness was recorded as not assessed by the LLM only (*n* = 13), clinician only (*n* = 2), or both evaluators (*n* = 2). Significant post-bronchodilator responsiveness was treated as the positive outcome. Values are point estimates (95% CI); confidence intervals were calculated using the Wilson score method.

## Discussion

The main finding of the present study is that a general-purpose LLM demonstrated variable performance across the main components of spirometry assessment. The model showed high sensitivity for individual technical quality criteria and high specificity for post-bronchodilator responsiveness. However, its performance for overall spirometry acceptability and multicategory interpretation was more limited. These findings suggest that publicly available LLMs may have an adjunctive role in spirometry quality assurance and clinical decision support, but they are not yet sufficiently reliable for independent interpretation of pulmonary function tests.

The assessment of spirometry acceptability and repeatability is a critical prerequisite for valid interpretation and is strongly recommended by ATS/ERS standards (6). It is therefore routinely performed in the PFT laboratories, although it requires appropriate training and clinical experience. The subjective nature of spirometry quality assessment may contribute to considerable inter-evaluators variability, even among experienced clinicians, highlighting the challenges of establishing a consistent reference standard (21). AI-based systems, including rule-based systems, machine learning models, deep learning models, LLMs, and multimodal foundations models have the potential to overcome these challenges.

The present study evaluated the ability of a widely available public LLM to assess the validity of spirometry tests. The present data demonstrate good overall performance in spirometry quality evaluation, achieving accuracy of 87% with excellent sensitivity of the LLM in identifying acceptable tests and fair specificity in identifying unacceptable spirometry tests. However, it should be noted that the LLM performed well in only recognizing acceptable spirometry tests, while the limited number and variety of unacceptable tests preclude firm conclusions regarding its ability to identify poor-quality spirometry. The predominance of acceptable tests compared to unacceptable tests may inflate overall accuracy and results in greater precision in estimating performance for acceptable tests than for the much smaller unacceptable tests. Consequently, sensitivity and specificity, particularly estimates based on the minority group, should be interpreted cautiously. Therefore, further validation using larger, more balanced datasets that include a broader spectrum of unacceptable spirometry tests is warranted.

The present findings are broadly consistent with previous studies evaluating artificial intelligence for spirometry quality assurance. Das et al. (2020) developed a convolutional neural network trained to determine the acceptability and usability of spirometry tests, applying ATS/ERS 2005 criteria to curves from the National Health and Nutrition Examination Survey. The model achieved an accuracy of 87% for acceptability and 92% for usability, with a sensitivity of 92% and a specificity of 96% for usability. Importantly, the system outperformed standard rule-based algorithm (15). Similarly, Wang et al. (2022) reported high accuracy for the assessment of FVC and FEV_1_ acceptability using a deep learning model incorporating spirometric indices and flow-volume curves morphology (17). Topole et al. (2023) found that AI-based systems performance was comparable to the experts’ performance in evaluating spirometry tests quality in asthma and chronic obstructive pulmonary disease (21). Velickovski et al. (2018) concluded that AI-based quality assurance systems may provide a feasible adjunct to spirometry quality control by improving consistency, reducing variability, and providing real-time feedback during testing (16).

A recent systematic review also concluded that AI-based systems are promising tools for spirometry quality assurance and may improve the assessment of maneuver acceptability while reducing the inherited evaluator-related subjectivity. However, the authors noted that the current evidence supporting AI-based spirometry quality assurance remains limited, largely due to methodological heterogeneity across studies and the absence of large-scale multicenter investigations to validate these systems in diverse clinical settings (18).

The performance of LLM in spirometry interpretation was less favorable than its performance in technical quality assessment. The present data show that LLM demonstrated moderate agreement between the LLM and the clinicians. Class-specific performance varied significantly; the LLM yielded its highest F1-scores for normal (0.81), but performed poorly in identifying obstructive defects (0.39). Previous studies of purpose-built artificial intelligence systems have reported stronger performance for pulmonary function test interpretation. Topalovic et al. (2019) developed and trained AI-based system to perform full PFT interpretation. The authors found that the model was able to correctly identify the diagnosis on 82% of the cases, significantly outperforming pulmonologists (22). Sunjaya et al. (2025) reported that an AI-based system achieved accuracy of 85% in identifying COPD in a cohort of 1,113 patients from primary care settings. The authors observed better performance in identifying COPD compared with other respiratory diseases (23). Similarly, a decision tree-based AI model evaluated 440 patients achieved an accuracy of 86% for PFT interpretation (24).

Evidence concerning general-purpose LLMs for spirometry and pulmonary function test interpretation remains limited and heterogeneous. Park et al. (2024) compared Gemini 1.5 Pro, GPT-4o, and Claude 3.5 Sonnet using portable spirometer data and found that classification performance varied across models and prompting strategies, with the highest accuracy achieved using few-shot prompting (25). Rohita et al. (2025) compared ChatGPT interpretations with those of respiratory physicians in 100 adult spirometry reports and reported high accuracy for distinguishing normal from abnormal spirometry, although disagreements were more apparent for obstructive and mixed patterns (26). In 100 pediatric spirometry tests, Özkaya et al. (2025) found moderate agreement between ChatGPT-4o and pulmonologists, with weaker performance for technical quality assessment and restrictive or mixed patterns (27). Abbas et al. (2026) evaluated ChatGPT using 10 complete pulmonary function tests and observed full agreement with pulmonologist interpretation in four cases, with discordance involving obstruction, restriction, bronchodilator responsiveness, hyperinflation or air trapping, and diffusing capacity (28). Collectively, these findings indicate that LLM performance varies according to the model, prompting strategy, input format, patient population, classification task, and reference-standard process, which may also contribute to differences between previous studies and the present findings.

Similar performance of ChatGPT was observed in electrocardiogram (ECG) interpretation. Günay et al. (2024) found that ChatGPT-4o demonstrated moderate agreement with cardiologists where ChatGPT-4 and Gemini advanced showed fair agreement. Cardiologists outperformed all evaluated LLMs. The authors concluded that while these models may serve as useful adjunctive tools for ECG interpretation and clinical decision support, they are not sufficiently accurate to replace clinicians’ judgment (29). Similar findings were reported by Zaboli et al. (2025), who found moderate agreement between ChatGPT and cardiologists for ECG interpretation. Nevertheless, cardiologists achieved superior interpretation accuracy. This has led the authors to conclude that ChatGPT should be used as an adjunctive rather than a replacement tool in clinical practice (30). Umit et al. (2026) reported that emergency medicine specialists outperformed customized ECG Reader – GPT in ECG interpretations. Emergency medicine specialists were able to interpret 77% of the ECG correctly while ECG Reader – GPT achieved only 24%, with poorer performance when interpreting complex ECGs (31). Collectively, these findings suggest that LLMs may provide clinical support but should not replace clinicians’ judgment.

Several factors may explain the moderate interpretation performance observed in the present study. Unlike AI-based systems which developed and trained for a specific purpose, publicly available LLMs such as ChatGPT-5.5 was designed to serve as general purpose language models. Therefore, they usually lack domain-specific training which is needed for highly specialized tasks. The domain-specific models are likely to optimize performance for specialized clinical tasks and achieve higher levels of accuracy, sensitivity, and specificity that are more suitable for clinical practice (32). Furthermore, the LLMs are designed mainly for language generation rather than the numerical reasoning. This makes them more susceptible to inaccurate calculation and threshold applications.

One possible explanation for the observed performance is the challenge of consistently applying the complex, sequential clinical algorithms required for spirometry interpretation. Another factor that may have contributed to the moderate performance is the inherent sensitivity of LLMs to prompt wording. Unlike deterministic clinical decision algorithms, LLMs can generate different responses to semantically similar prompts, potentially resulting in variability in spirometry interpretation despite identical input data (33). Although publicly available LLMs demonstrate promising capabilities, the current evidence is insufficient to support their independent use in pulmonary function tests’ interpretation. The observed moderate performance of LLM highlights the need for cautious implementation in clinical settings. They may serve as adjunctive educational or decision-support tools but should not replace expert interpretation of pulmonary function tests.

The bronchodilator responsiveness analysis showed more favorable performance. Among the 117 participants with valid paired classifications, the model achieved an accuracy of 0.97, specificity of 1.00, PPV of 1.00, and Cohen's *κ* of 0.86. Sensitivity was lower at 0.77. These findings suggest that the model accurately identified the absence of significant post-bronchodilator responsiveness in the analyzed sample and showed strong agreement with the clinician reference category. However, responsiveness assessment was unavailable in 17 participants because at least one classification was considered unacceptable or uninterpretable. Consequently, responsiveness performance should be interpreted in the context of the model's preceding quality assessment decisions.

The present investigation is not without limitations. First, the evaluated model (ChatGPT 5.5) was a general-purpose LLM rather than a domain-specific model specifically trained for spirometry interpretation. Consequently, the findings should not be generalized to all specialized AI systems optimized for pulmonary functions assessment purposes. Second, although standardized prompts were used throughout current investigation, LLM responses are known to be inherently sensitive to prompt design. Therefore, different prompt formulations may have influenced the interpretation accuracy, sensitivity and specificity. Third, the limited demographic characteristics and clinical diversity of the study population, which was predominantly Caucasian, relatively young, and small number of smokers, may restrict the generalizability of the current findings to broader patient populations. Finally, Although the reference standard consisted of interpretations made by the pulmonary team and represents the current clinical standard for spirometry interpretation, some degree of inter-observer variability is unavoidable. Moreover, LLM was limited to the information contained in the spirometry report, whereas clinicians may have incorporated additional clinical context into their interpretations.

In Summary, the present investigation suggests that general purpose LLM poses limited reliability to be considered as independent tool for spirometry interpretations. Clinically acceptable performance would likely require domain-specific training, rigorous external validation, and integration with standardized pulmonary functions interpretation algorithms before it become available for routine clinical use. Further investigations are warranted to confirm the present findings.

## Data Availability

The raw data supporting the conclusions of this article will be made available by the authors, without undue reservation.
